# Evaluation of Asphalt Mixtures Containing Metallic Fibers from Recycled Tires to Promote Crack-Healing

**DOI:** 10.3390/ma13245731

**Published:** 2020-12-16

**Authors:** Alvaro González, José Norambuena-Contreras, Lily Poulikakos, María José Varela, Jonathan Valderrama, Alexander Flisch, Martín Arraigada

**Affiliations:** 1 Department of Construction Engineering and Management, School of Engineering, Pontificia Universidad Católica de Chile, Avenida Vicuña Mackenna, Santiago 4860, Chile; j.valderrama@dictuc.cl; 2 LabMAT, Department of Civil and Environmental Engineering, University of Bío-Bío, Avenida Collao, Concepción 1202, Chile; jnorambuena@ubiobio.cl (J.N.-C.); mvarela@ubiobio.cl (M.J.V.); 3 Empa, Swiss Federal Laboratories for Materials Science and Technology, Überlandstrasse, 129 CH-Dübendorf, Switzerland; lily.poulikakos@empa.ch (L.P.); alexander.flisch@empa.ch (A.F.); martin.arraigada@empa.ch (M.A.)

**Keywords:** asphalt mixture, metallic fibers, waste tires, crack-healing, computed tomography.

## Abstract

This paper reports part of an international research project with the long-term aim of developing more sustainable asphalt mixture with crack-healing properties by the addition of recycled metallic waste from industrial sources. Specifically, this article presents an evaluation of the physical, thermophysical, and mechanical properties of asphalt mixtures with metallic fiber obtained from recycled tires for crack-healing purposes. Detailed results on the crack-healing of asphalt mixtures will be reported in a second article. Results showed a small reduction on the bulk density and increase in the air voids content was quantified with increasing fiber contents. The experimental results showed that mixing and compaction was more difficult for higher fiber contents due to less space for the bitumen to freely flow and fill the voids of the mixtures. Computed tomography (CT) results allowed to identify clustering and orientation of the fibers. The samples were electrically conductive, and the electrical resistivity decreased with the increase of the fiber content. Fiber content had a direct effect on the indirect tensile stiffness modulus (ITSM) and strength (ITS) that decreased with increasing temperature for mixtures and with increase in fiber content. However, the indirect tensile strength ratio (ITSR) was within acceptable limits. In short, results indicate that fibers from recycled tires have a potential for use within asphalt mixtures to promote crack-healing.

## 1. Introduction

Tire production for vehicles and trucks is globally increasing given the growing population, economy, and transportation. Worldwide, tire production exceeds 2.9 billion units per year [1]. It is estimated that for every tire placed in the market, another tire reaches its service life and becomes waste [2]. This massive amount of waste occupies a large area in landfills and causes environmental hazards. Waste tires are nondegradable due to cross-linking of vulcanized rubber with sulfur bonds, and the addition of antioxidants and antiozonants during tire production [3]. Burning or using tire as fuel may produce toxic gases that are harmful for the environment and may cause destructive pollution of natural air [4,5]. Tire piles often provide breeding grounds for pests and insects such as mosquitoes, because their shape and impermeability allow them to hold water for extended periods, facilitating the spreading of contagious diseases [6]. Different environmental policies, like extended producer responsibility, control waste tire disposal in developed countries have aimed to address this problem. However, waste tires are still landfilled in many countries causing major public health risks and environmental issues [7].

Common components of tires are natural and synthetic rubbers, carbon black, metallic fiber, textile fabric, and additives [8]. Rubber from recycled tires has many applications in the industry. Tire pyrolysis, i.e. the thermal degradation of waste tire at elevated temperature, produces value-added products such as tire pyrolysis oil, pyro char, pyro gas, which are used as alternative fuel for engines [6,9]. Rubber from recycled tires are a source of sustainable new material that are also used to create environmentally friendly and low-cost composites [10]. In civil engineering materials, researchers have used rubber from recycled tires in mortars [5]; cement concrete [2,4,11]; geogrids in granular soils [12]; aggregates [13,14]; soil reinforcement [15]; composite membranes [16,17]; and asphalt [18,19,20]. The use of metal fiber waste from tires is less explored; however, researchers have investigated the effect of fiber waste from tires in the mechanical, fire resistance, and acoustic properties of concrete [21,22,23,24,25,26]. The use of metal fiber waste from tires in asphalt mixtures is less explored than concrete, with only few studies found in the literature [27]. Nevertheless, researchers have used different types of fiber mainly to increase the mechanical performance and durability of asphalt mixtures. Some of these fibers are products especially fabricated with the main purpose of material reinforcement [28,29,30], while others are waste byproducts from various industries [31].

In addition to the enhancement of the mechanical properties of asphalt mixtures, metallic fiber or particles from virgin materials or waste improve the electrical and thermophysical properties of asphalt mixtures [32]. This enhancement has led to the development of innovative materials, like pavements with crack-healing properties by external heating [33,34,35,36]. In these mixtures, metals, normally steel wool fibers, are added because they conduct and absorb more thermal energy than bitumen and aggregates, increasing the electrical conductivity of the asphalt mixtures [37]. To increase the temperature and heal this type of asphalt mixture, an external electromagnetic field, such as those applied by electromagnetic induction or microwaves, is artificially applied to heat the fiber. Later, the fiber heat transfers to the rest of the mixture, reducing the bitumen viscosity, making the bitumen flow and seal, repairing open cracks.

Only a few studies have included waste materials for the preparation of asphalt mixtures with crack-healing purposes. Norambuena-Contreras et al. [38] evaluated the effect of metallic waste content on the thermophysical, electrical, and microwave crack-healing properties of asphalt mixtures, adding two types of metallic waste in different contents: steel wool fiber and steel shavings from the metal turnery industry. Gonzalez et al. [39] evaluated the effect of adding metal shavings and reclaimed asphalt pavement (RAP) on the properties of asphalt mixtures with crack-healing capabilities by microwave heating, concluding that asphalt mixtures with RAP and waste metal shavings have the potential of being crack-healed by microwave heating. The researchers have also compared different type of metal waste and other type of ceramic additives in the microwave crack-healing of asphalt mixtures [40]. Although a good number of articles on the crack-healing of asphalt mixtures with fibers or metallic waste are found in the literature, metallic fibers from recycled tires have been sparsely considered for this purpose [41].

This paper reports the first part of an international research project between Chile and Switzerland, with the long-term aim of developing a sustainable asphalt mixture with advanced mechanical, thermophysical, and crack-healing properties by the addition of recycled metallic waste from industrial sources. The objective of the paper is to evaluate the physical, thermophysical, and mechanical properties of asphalt mixtures with crack-healing potential containing different amounts of metallic fiber obtained from recycled tires. Detailed results on the crack-healing of asphalt mixtures will be reported in a second article. Asphalt mixture with one type of metallic fiber, bitumen, and aggregates, were studied by experimental tests. The results indicate that fibers from recycled tires have a positive effect on the properties of asphalt mixtures, as further discussed below. In addition, results indicate that fibers from recycled tires have a potential for use within asphalt mixtures to promote crack-healing.

## 2. Materials and Methods

### 2.1. Raw Materials

The aggregates, sourced from a local construction company located in Santiago, Chile, were provided in different fractions and later combined to produce the target particle size distribution of a dense asphalt mixture. The aggregates supplied consisted of a 19 mm coarse gravel, a 12.5 mm gravel, and a crushed dust (Table 1). These fractions were blended to achieve the particle size distribution of dense asphalt mixtures. The penetration grade of the CA24 bitumen used for mixture preparation was 80/100 mm at 25 °C and its absolute viscosity >2400 poise at 60 °C. The bitumen content targeted for all the asphalt mixtures was 5.2%, by mass. The virgin metallic fibers with rubber scraps were obtained from a tire recycling plant from Tire Recycling Solutions, Préverenges, Switzerland.

### 2.2. Preparation of Asphalt Mixture Specimens

The aggregates and bitumen were heated in the oven for a minimum of two hours at 150 °C before mixing. The mixing order for specimens with metallic fibers followed these steps:

- The bitumen was placed in a metallic container previously heated and used for the mixing to keep the mixing temperature constant.
- Metallic fibers from recycled tires with rubber scraps bounded were initially cleaned in a solvent solution for a few hours and then were heated to 500 °C to improve the ductility of the fibers during the mixing and compaction.
- Then, approximately 1% of the metallic fibers previously heated were blended with the mixture. The remaining metallic fibers were gradually included after the addition of aggregate lots.
- Small lots of aggregates were added to the mixtures, separated by particle size. The lot with the largest particles was the first to be added.
- Once the aggregates were entirely covered with bitumen, the next lot with smaller particles was added to the mixture.

Marshall specimens were produced taking mixture from each batch (diameter = 100 mm; height = 60 mm, approximately). A sample of approximately 1150–1200 g of mixture was used for the preparation of each specimen. The sample was compacted using a Marshall hammer (Humboldt, IL, USA) giving 75 blows to each specimen face. The estimated bitumen content for each Marshall specimen was 58 g. The metallic fiber contents added to each specimen were 1.5%, 2.5%, and 3.5% of the bitumen volume, equivalent to 7 g, 11.6 g, and 16.3 g, respectively. In total, 117 Marshall test specimens were prepared.

### 2.3. Characterization of Metallic Fiber from Recycled Tires

**Figure 1:**
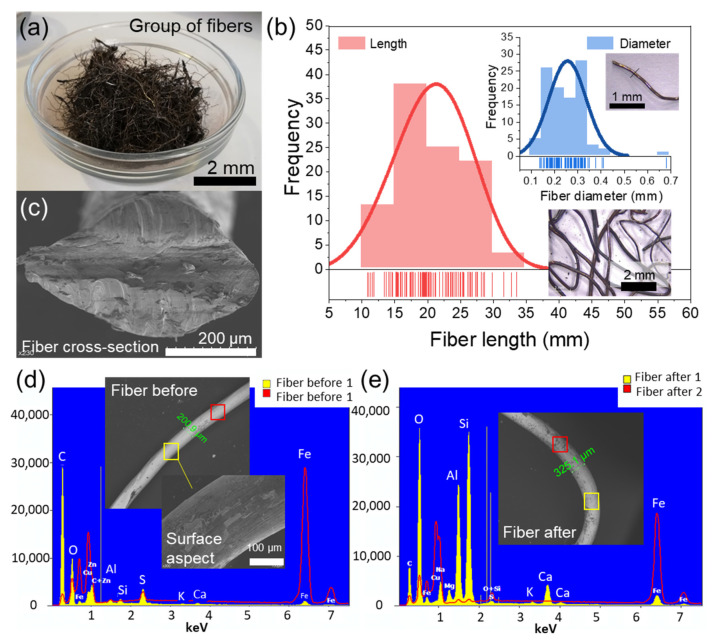
Characterization of metallic fiber from recycled tires: (**a**) group of fibers; (**b**) ESEM image of the cross-section of the cutting fiber; (**c**) frequency histogram of the length and diameter of fibers data with Weibull and normal fitting respectively; (**d**) ESEM-EDX elemental analysis of the fibers before and; (**e**) after manufacturing indicates more mineral residue and oxidation after the mixing process.

Size, surface aspect and cross-section of the metallic fibers from recycled tires were characterized by optical and environmental scanning electron microscopy (ESEM), respectively following the methodology described by Norambuena-Contreras et al [38]. To this end, one hundred metallic fibers were randomly selected. Later, the diameter and length of each fiber was determined by taking photographs under a stereoscopic microscope (Leica EZ4, Wetzlar, Germany) with 35× magnification and measured with the image processing software ImageJ^®^ (version IJ 1.46r, Bethesda, MD, USA). An image of the metallic fibers from recycled tires used and the frequency histogram of their length and diameter are shown in Figure 1a,b, respectively.

Moreover, the surface aspect ratio and cross-section of metallic fibers (see Figure 1c) randomly selected was observed by using a ESEM Quanta FEG650 (ThermoFisher, Waltham, MA, USA) in the high vacuum mode. Additionally, metallic fibers were characterized through ESEM-EDX analysis before the mixing and compacting processes. To do it, asphalt mixture sample with 2.5% fibers was placed in a toluene solution for a few hours and then the solution was filtered for that fibers were extracted using a magnet. The ESEM images are a result of the interaction of the electron beam with the atoms of the sample. The higher the atomic number the brighter the images. Energy dispersive X-rays (EDX) are characteristic X-rays that are produced due to displacement of electrons in the sample shells. In short, it was determined that metallic fibers from recycled tires had an average diameter of 0.256 mm (standard deviation (SD) = 0.079 mm), see Figure 1b,c with an average aspect ratio of 81, and an initial length range of 11–40 mm, see histogram in Figure 1b, which indicates that both short and long metallic fibers were randomly added to the asphalt mixture. Likewise, after the manufacturing process, the main difference between fibers is that there are particles of minerals attached to the fibers as a result of the mixing with aggregates. EDX analysis at two locations on the fibers is shown in Figure 1d,e showing that after the mixing process there is more mineral residue and oxidation in the fibers.

### 2.4. Physical Characterization of Asphalt Test Samples

Two important physical properties of asphalt mixtures are bulk density and air voids content. Bulk density is the ratio between the dry weight and the real volume of each specimen. The real volume of each specimen is determined using the weight of the water-submerged specimen. Since dry weight is known, bulk density (*ρ_b_*) is calculated for each asphalt specimen. The material composition for and their density for each asphalt mixture type were known, hence the theoretical maximum density for each specimen was found (*ρ_max_*). Therefore, the air voids content (AV) of each test sample was calculated as [42]:

(1) $$AV=\frac{\rho_{max}-\rho_{b}}{\rho_{max}}$$

Six test specimens were used for the calculation of the average bulk density and air voids content. 

### 2.5. Fibers Distribution and Orientation by X-Ray Computed Tomography

Depending on the amount of fiber used, clustering can increase the material’s inhomogeneity. This in turn can reduce its mechanical performance and affect the homogeneous distribution of the induced heat, as the fibers will be concentrated in some areas and will not be present in others. Furthermore, the direction in which the fibers are mostly aligned might influence the isotropy of the material. Therefore, X-ray computed tomography (CT) was used to analyze the distribution and orientation of the fibers of the three studied concentrations (1.5%, 2.5% and 3.5% of fibers in bitumen volume). Since X-rays were not able to penetrate the mass of the Marshall specimens (100 mm diameter), cylinders of approximately 40 mm diameter and 40 mm height were cored from the center of the 100mm specimens and used as samples for the scans.

The CT system used is a RayScan500 (RayScan, Meersburg, Germany) equipped with a 300 kV micro focus X-ray source FineTec FORE 300.01Y RT (PerkinElmer, Waltham, MA, US) and a digital X-ray detector PerkinElmer XRD 1611 CP3 (PerkinElmer, Massachusetts, US). The detector is based on a 16″ amorphous silicon sensor operating as a two-dimensional photodiode array. X-rays are converted into light using a CsI (Ti) scintillator with needles directly deposited on the photodiodes. The information is digitized in 16 bits to achieve a high dynamic range. The detector has a pixel size of 100 µm and an image size of 4096 × 4096 pixels. The operator of the CT system has three longitudinal and one rotational axis and is able to handle samples with a weight up to 50 kg. The best achievable spatial resolution of the CT system is about 3 µm. The asphalt samples were scanned at X-ray source settings of 290 kV/490 µA. A total number of 1260 X-ray projections were acquired with an integration time of 999 ms and a detector pixel binning of 2. The source–detector distance of the scanner was set to 1534.9 mm and the source–object distance was 445.5 mm.

In this study, the reconstructed three-dimensional (3D) images of each measurement achieved a resolution of (116 µm)^3^, i.e. this is the size of the smallest cubic units in the images called voxels. For visualization purposes, each voxel is assigned a gray tonality proportional to the X-ray attenuation coefficient, which in turn depends on the material’s density. Low-density materials are weak X-ray absorbers and exhibit low attenuation. When visualized on a typical gray-scale map these voxels appear as dark regions. On the other hand, high-density materials appear as brighter regions [43]. In order to perform the analysis of the fibers’ distribution and orientation it is first necessary to separate them from the aggregates, bitumen and void matrix through an image segmentation that splits the voxels according to the material. As described earlier, the 16 bit image digitalization corresponds to attenuation coefficients that range from 1 to 65,536 and can be represented as a histogram of gray-scale intensity distribution. Ideally, each material composing the asphalt concrete samples should present a clear peak in the histogram and segmentation could be easily performed by setting global thresholds between these peaks. However, due to several reasons like weak contrast between materials, noise or so-called partial volume effects, voxels often show attenuation coefficients different to those expected for a given material [44]. Thus, to avoid errors in the segmentation, the processing method should be carefully selected through a sensitivity analysis and validated against a known parameter. In this study, the volume of fibers calculated from the CT scan images was compared to the real volume of fibers in order to assess the quality of each methodology. To that end, the fibers contained in the scanned samples were recovered dissolving the asphalt binder with toluene. Fibers and aggregates were separated manually using sieves and magnets. Then, the weight of the fibers was measured to obtain their volume.

The processing of the CT scans images was achieved with the software VGstudio Max 3.3 (VolumeGraphics, Heidelberg, Germany) [45]. This software offers the possibility of using a segmentation tool for the analysis of defects like pores or inclusions, considering two algorithms. The ‘Only threshold’ algorithm is based on a global gray threshold in which each voxel is examined and defined as pore or inclusion if it is below or above a certain value respectively. On the other hand, the *VGDefX* is a more sophisticated algorithm that checks if some voxels, considered as seeds, are defects. Then, it looks in the neighborhood for other voxels that are part of the defect following a certain probability criteria. This algorithm allows for gray value variations and includes noise reduction for seed point location. In this work, the fibers were assumed as inclusions in the asphalt matrix, as metal has higher density than bitumen or stones and could be differentiated through its gray value. It has to be mentioned that, due to the fact that the fiber’s diameter is close to the image resolution, blurred fibers edges can cause partial volume effects with a relatively big impact on the segmentation. Therefore, to perform the segmentation the most precise *VGDefX’* algorithm was selected. As part of the sensitivity analysis, different threshold values were manually chosen based on the histogram of gray-scale intensity. The volume of the fibers was then calculated. However, it was found that the aggregates also have metallic inclusions with similar gray values as the fibers that cannot be separated only with the segmentation. Thanks to a tool from VGstudio Max 3.3 [45], selected inclusions can be filtered by their geometric features. Fibers have an elongated shape that differ completely from the typical round form of the inclusions in aggregates, which are also usually of small size. Therefore, three features were used to identify which of the inclusions correspond to fibers:

- Size: amount of voxels that are part of the inclusion. Considering the average thickness and length measured in last section, the fibers have an average diameter and length of ca. 0.256 mm and 20 mm respectively, which makes a volume of ca. 1 mm^3^. Values that differ too much of this volume are filtered out, as explained in Section 3.2.
- Sphericity: ratio between the surface of a sphere with the same volume as the inclusion and the surface of the defect, with values between 0 and 1 being 1 a perfect sphere. Fibers have values towards 0.
- Compactness: ratio between the volume of the defect and the volume of the circumscribed sphere, with values between 0 and 1, being 1 a perfect sphere. Compactness of fibers are close to 0.

**Figure 2:**
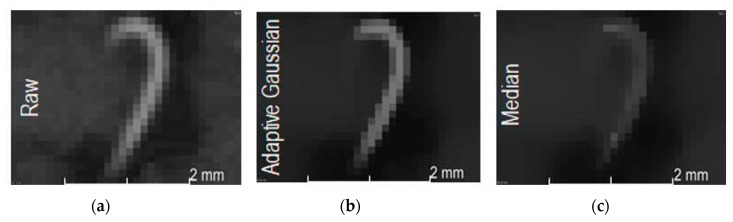
2D slices showing (**a**) a raw version (**b**) the effect of the adaptive Gaussian and (**c**) median filters on the image of one fiber.

In order to reduce white noise and enhance the quality of the images before segmentation, the software offers the possibility to apply different filters. According to the literature [46], median and Gaussian filters available in VGstudio Max 3.3 [45] exhibit good performance in reducing white noise and greyscale value outliers but they might weaken the contrast in the edges of the fibers and induce a bias in the gray scale values. A visual assessment of the use of these filters is presented in Figure 2. Although the adaptive Gaussian filter seems to provide a better contrast between the fiber and the surrounding bitumen-aggregate matrix, the fiber size appears to be reduced. On the other hand, a blurred image is the consequence of the application of a median filter. Consequently, in this work no image filter was applied.

**Figure 3:**
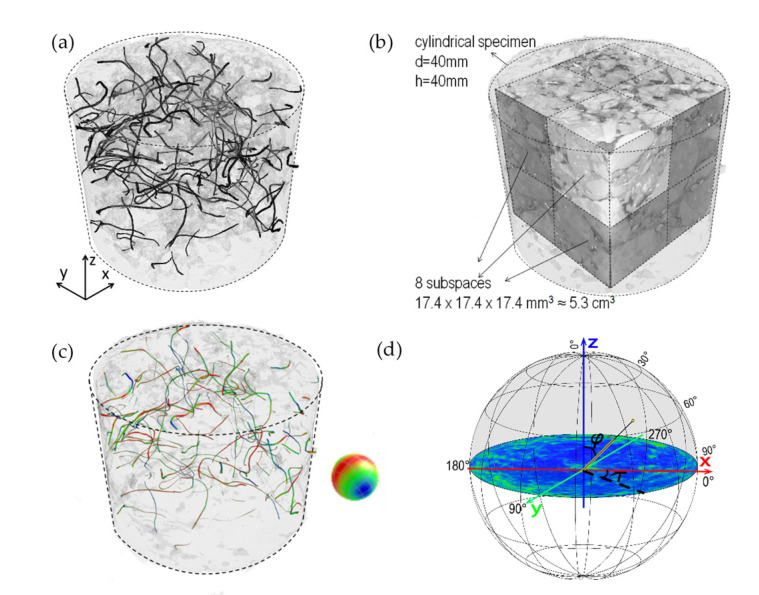
(**a**) Aspect of the specimen with 1.5% of fibers. (**b**) View of the subspaces used for the distribution analysis. (**c**) Space orientation of the fibers, according to the color mapping shown in the sphere. (**d**) Schematic of the construction of a polar plot for fiber orientation analysis.

Once the sensitivity analysis was performed and the best segmentation selected (see Figure 3), the distribution of the fibers was analyzed in order to determine possible accumulation in clusters. The specimen 3D image was divided into eight cubic subspaces with a side length of 150 voxels (ca. 17.4 mm), equivalent to almost 5.3 cm^3^ (see Figure 3b). For each subspace, the percentage of fibers was calculated by adding up all the segmented voxels assigned as fibers, divided by the defined total volume of the subspace. The comparison of the percentage of fibers in each subspace was used as an indication about possible clusters. Based on the same segmentation method, an analysis of the orientation of the fibers was carried out using another tool in the software VGstudio Max 3.3 [45], the fiber composite material analysis (FCMA) module. This tool can present the orientation of the fibers based on a spatial coding visualized on a color sphere, as displayed in Figure 3c. To quantify if there is a direction or a plane to which the fibers are mostly aligned, each orientation is projected to a sphere as a histogram where the frequency is displayed as a color (see Figure 3d). The polar plot shown later in the results section, shows the surface of the half-sphere unrolled into 2D. The center of the plot represent the fibers aligned vertically, coincident with direction of movement of the Marshall hammer used for compaction of the specimen (polar angle φ = 0°). Instead, the edge of the polar plot is perpendicular to the compaction (polar angle φ = 90°). The azimuth angle τ represent the orientation of the fiber in horizontal plane and is less relevant in the context of the compaction method.

### 2.6. Electrical and Thermal Properties of Asphalt Test Samples

Electrical resistivity and thermal conductivity of the asphalt mixture test samples without, and with, different metallic fiber contents were measured following the experimental procedures described by Norambuena-Contreras et al. [38]. Firstly, to measure the electrical resistance of each asphalt test sample a megohmmeter device (IR4056-20, Hioki, Nagano, Japan) connected to two stainless steel electrode plates with dimensions 10 cm × 15 cm was used. The electrodes were placed on the opposite faces of the test samples and a pressure of 1 kPa was applied on them, ensuring that the samples were centered, and the plates horizontally aligned during the measurements. After that, electrical resistance measurement of test samples was recorded, and their electrical resistivity was calculated applying Ohm’s law [47]:

(2) $$\rho =\frac{R\cdot S}{l},$$

where *R* is the electrical resistance of each test sample in Ω; *S* is the electrode plate area in m^2^; and *l* is the thickness of each asphalt sample in m. The electrical resistivity of each sample tested was determined as the average of three tests.

Additionally, the thermal conductivity of each asphalt test sample was measured using the thermal needle probe method, based on the transient linear heat source theory. To this end, KD2-Pro thermal analyzer (Decagon Devices, Pullman, WA, USA) device with a handheld controller and a needle thermal sensor (RK-1, Decagon Devices, Pullman, WA, USA) (in the range of 0.1–6.0 Wm^−1^K^−1^) was used. To carry out the tests, the thermal sensor covered with a polysynthetic thermal compound was embedded in the test sample that was previously drilled. During the measurement, the tested sample was placed on two other test samples to ensure adiabatic conditions. The duration of each was 10 min. Lastly, for each test sample, the thermal conductivity (λ) was calculated using the following relationship [47]:

(3) $$\lambda =\frac{q}{4\cdot \pi \cdot m},$$

where *q* is the heat generated by the sensor in W/m; m is the slope of the linear relationship between the temperature range for the testing time; and *λ* is the thermal conductivity of each asphalt test sample in Wm^−1^K^−1^. The thermal conductivity of each test sample was determined as the average of three measurements.

### 2.7. Stiffness Modulus and Indirect Tensile Strength

The stiffness was measured using the indirect tensile stiffness modulus test (ITSM), following the European Standard UNE-EN 12697-26:2006 Annex C [48]. In the test, a vertical repetitive load is applied to the vertical diameter of the Marshall cylindrical specimen. The applied load deforms the specimen, reaching a 50 εμ horizontal deformation measured using two linear variable differential transducers (LVDTs) located parallel to the horizontal diameter. Ten repetitive loading pulsations were applied on two orthogonal diameters to measure the potential anisotropy of the material. For the calculation of the ITSM the following equation was used [42,49]:

(4) $$ITSM=\frac{P\cdot \left(0.27+\nu \right)}{d\cdot t},$$

where *P* is the peak vertical load (in N) diametrically applied, *ν* is the Poisson’s ratio (assumed 0.35 based on [48] findings), *d* is the horizontal maximum deformation (in mm), *t* is the specimen height or thickness (in mm), and *ITSM* is the calculated stiffness modulus (in MPa). The stiffness modulus tests were performed in specimens with metal fiber contents of 0%, 1.5%, 2.5%, and 3.5%. The tests were carried out in a temperature-controlled chamber at temperatures of 5, 20, 30, and 40 °C. To ensure that the aim temperature was uniform in all the specimen volume, before testing the specimens were left in the temperature chamber for a minimum of four hours.

The indirect tensile strength (ITS) test was conducted on Marshall specimens loaded vertically through its diameter at a constant speed of 5 ± 0.2 mm/min. The compressive load induces transverse tension stress on the central portion through plane load and horizontal tension. The horizontal stress induced on the center of the specimen is calculated as follows [42,50]:

(5) $$\sigma_{max}=\frac{2\times F_{max}}{\pi \times D\times t}$$

where *σ_h_* is the maximum tensile stress in the center of the specimen (N/mm^2^), *F_max_* is the maximum force (N), *D* is the specimen diameter (mm), and *t* is the specimen height (mm). Marshall specimens for the ITS dry test were conditioned and tested at 20 °C. Marshall specimens for the ITS wet test were first soaked in water at 40 °C for 72 h and later tested at 20 °C.

## 3. Results and Discussion

### 3.1. Effect of the Metallic Fiber from Recycled Tyres on the Physical Properties of Asphalt Mixtures

**Figure 4:**
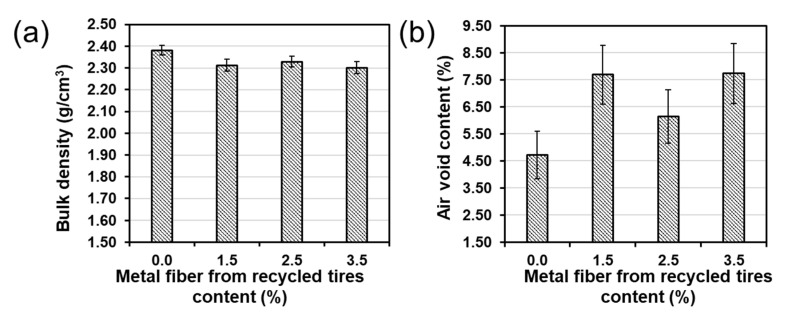
Metal fiber effect from recycled tires on the (**a**) bulk density; and (**b**) air voids content.

The average bulk density for all specimens tested was 2.331 g/cm^3^ (SD = 0.036 g/cm^3^). The general effect of the metal fiber from recycled tires content was a small reduction on the bulk density, with average bulk densities of 2.382, 2.312, 2.329, and 2.301 g/cm^3^ for metal fiber contents of 0%, 1.5%, 2.5%, and 3.5%, respectively (Figure 4a). The general average air voids for mixtures with different fiber contents was 6.6%. Results show an overall air voids content increase with increasing fiber contents. The average air voids measured were 4.7%, 7.7%, 6.1%, and 7.7%, for metal fibers contents of 0%, 1.5%, 2.5%, and 3.5% (Figure 4b).

The dissimilar bulk density and air voids are explained by the different content of metal fibers, while the aggregates and bitumen content remained constant in the mixtures. The higher air voids with increasing metal fiber content is explained by the formation of metal fibers clusters in the mixture, as will be shown later in this paper. This result is consistent with observations made during mixing and compaction in the specimen preparation. The mixing and compaction in mixtures with higher fiber contents was more difficult because there is less space for the bitumen to easily flow. Hence, for the bitumen is more difficult to fill the voids of the mixtures.

### 3.2. Fibers Distribution and Orientation of the Metallic Fibers into the Asphalt Mixtures

**Figure 5:**
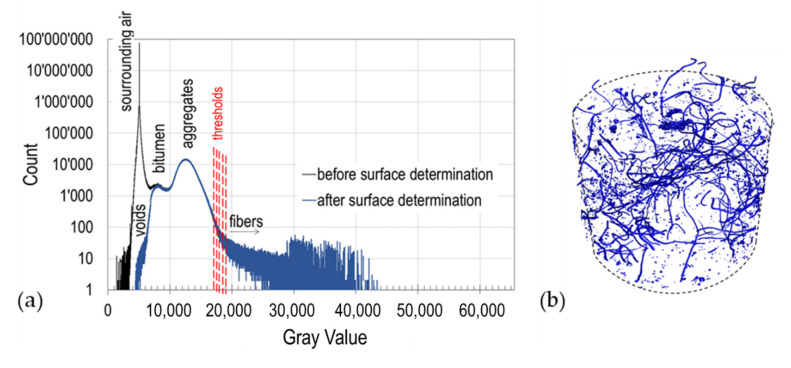
(**a**) Histogram of grayscale distribution of the images of the 1.5% specimen before and after surface determination showing the materials peaks and the threshold selected. (**b**) View of the inclusions after segmentation (no filter), 1.5% fiber content.

**Figure 6:**
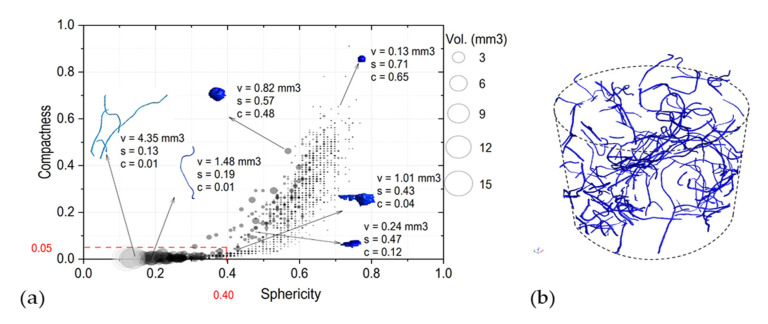
(**a**) Overview the geometrical parameters of the inclusions, in red the values set to filter out the aggregate’s inclusions. (**b**) View of the inclusions after segmentation and filtering.

**Figure 7:**
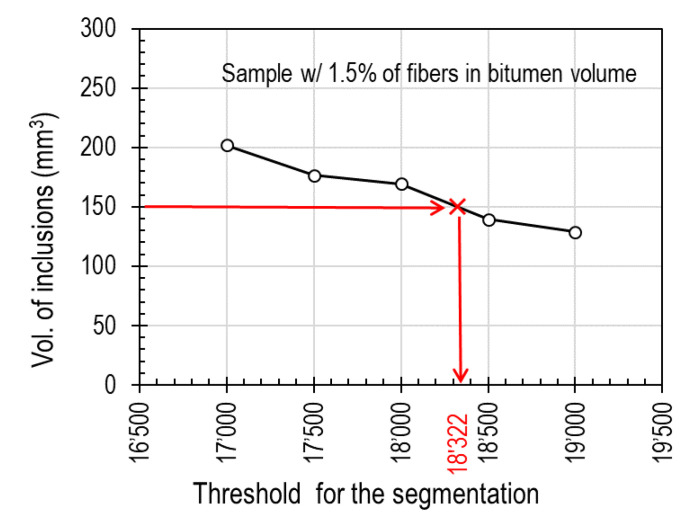
Sensitivity of the selected threshold on the volume of fibers and selection of the “right” threshold based on the volume of fibers recovered from the sample.

The sensitivity to the segmentation method was studied on volumes defined using the advance surface determination tool of VGstudio MAX 3.3, which stablishes a boundary between air and the specimen through their gray values. Figure 5a shows, as an example, the histograms of the distribution of gray values for the specimen containing 1.5% of fibers, before and after surface determination. It is possible to observe peaks for the air, bitumen and aggregates, but not for the fibers as their gray values are blended with the aggregate’s values. Only by setting specific thresholds it is evident which region of the histogram represents the fibers. As explained in Section 2.5, different thresholds values (for example 17’000, 17’500, 18’000, 18’500 and 19’000 for the 1.5% specimen) were proposed for the *VGDefX* method. These values were selected based on a visual assessment of how they affect segmentation. Figure 5b displays the inclusions obtained using the 18’000 threshold that contain not only the fibers but also some metallic particles of the aggregates. As detailed in Section 2.6, fibers and aggregate inclusions differ up to some degree in their size and shape. This can be observed in Figure 6a, where all 2602 resulting inclusions are plotted regarding their sphericity, compactness and size. By setting thresholds in these parameters it is possible to filter out most of the aggregates’ inclusions and only leave the fibers for analysis. It was observed that all fibers have sizes above 0.1 mm^3^ and sphericity and compactness values below 0.39 and 0.05 respectively. Nevertheless, any aggregate inclusion outliers still present after filtering were manually erased after visual assessment, obtaining the final distribution of fibers (see Figure 6b). In Figure 7, the fibers’ volumes obtained after segmentation and filtering are plotted against the selected threshold. For the sample containing 1.5% of fibers, 1.2 g of fibers were recovered, which represents a volume of 150 mm^3^. Using Figure 7 it is possible to interpolate and obtain the threshold (18’322) that would match the real volume of fibers, which was then used for the final segmentation. The same method was used to analyze the 2.5% and 3.5% fiber samples.

**Figure 8:**
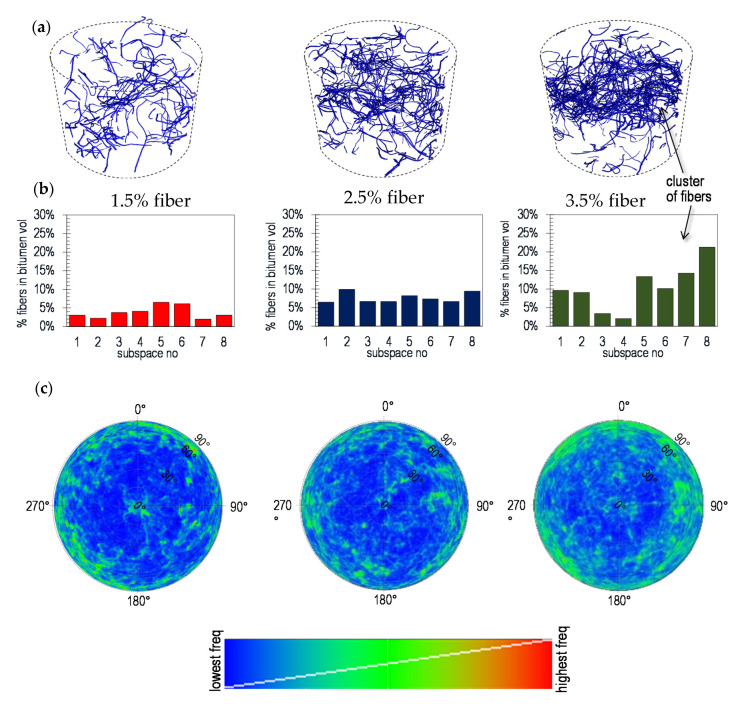
(**a**) 3D view of the fibers after segmentation. (**b**) Volume of fibers for each subspace. (**c**) Polar plot showing the orientation of the fibers. The colors show the frequencies of the fiber orientations.

The columns in Figure 8 present the analysis of distribution and orientation of the fibers for a fiber concentration of 1.5%, 2.5% and 3.5% by volume of bitumen. The subfigures presented in the first row (Figure 8a) show a 3D vision of the fibers inside the cylindrical samples. Although they already give an indication about the formation of possible clusters, in the second row (Figure 8b) the amount of fibers per subspace is presented as charts. Similar heights in the columns of the chart indicate a homogenous distribution of the fibers. Conversely, a poor fiber distribution concentrates the fibers in specific spots of the sample forming clusters. As a result, the columns of the chart should present large differences, with the highest values representing clusters. This is the case of the sample with 3.5% of fibers, in which the subspace 4 has the smallest concentration and the subspace 8 the highest. It is then evident that a cluster was formed on the top of the sample. This might indicate that using more than 2.5% of fibers might produce clusters. The orientation of the fibers is represented by a polar plot in Figure 8c. The colors show the frequencies of the orientations projected on a sphere, with blue and red as the least and most frequent orientations respectively. From the color distribution it can be observed that the fibers tend to orient towards a horizontal plane, i.e., perpendicular to the compacting force with the intensity increasing with fiber content.

### 3.3. Effect of the Metallic Waste on the Electrical and Thermal Properties of Asphalt Mixtures

**Figure 9:**
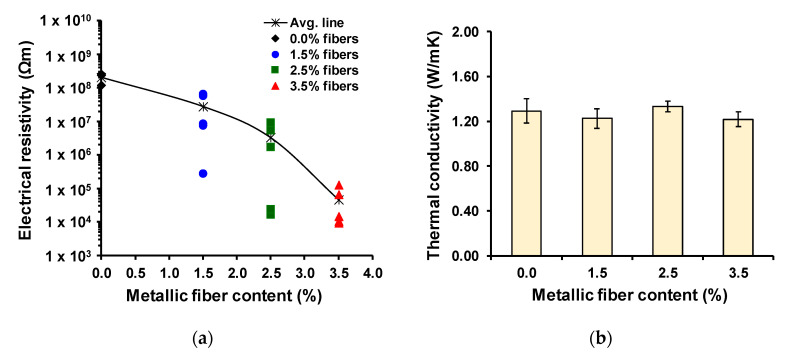
Results of (**a**) electrical resistivity and (**b**) thermal conductivity of the asphalt test samples.

Figure 9a shows all the electrical resistivity results measured on test samples without, and with, different metallic fiber contents. The average electrical resistivity for the reference mixture was 2.05 × 10^8^ Ωm, while the average electrical resistivity for test samples with 1.5%, 2.5% and 3.5% of fiber content was 2.76 × 10^7^ Ωm, 3.22 × 10^6^ Ωm and 4.59 × 10^4^ Ωm, respectively. Results show that (1) samples with metallic fibers presented an electrically conductive behavior, and (2) the average electrical resistivity decreased with the increase of the fiber content (Figure 9a), thus increasing the electrical conductivity of samples, consequent with the percolation theory on electrically conductive composites [36]. In summary, with the range of fiber amounts used in this study from 1.5% to 3.5%, the percolation point, threshold value from where the electrical resistivity remains constant with the content, was not observed. In addition, Figure 9b shows the average thermal conductivity results and error bars equivalent to one standard deviation. Thermal conductivity was measured on asphalt test samples without, and with, different metallic fiber contents. Figure 9b shows that, in general, the thermal conductivity decreases with the addition of metallic fibers with respect to the reference mixture; however, this reduction was not significant. The mixtures without fibers showed the highest average thermal conductivity with a value of 1.293 Wm^−1^K^−1^ (SD = 0.106 Wm^−1^K^−1^). This result was previously explained by Norambuena-Contreras et al., who related the air voids content increase with the addition of metallic waste to the asphalt mixtures. Accordingly, the increase of air voids dissipates the heat in the mixtures, reducing the heat transmission of the mixtures [38]. 

### 3.4. Effect of the Metallic Waste on the Mechanical Properties of Asphalt Mixtures

**Figure 10:**
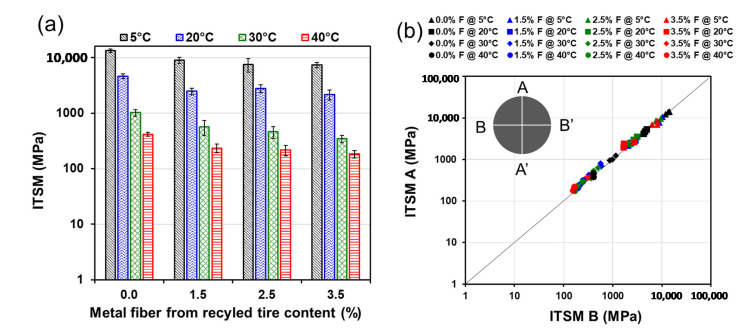
Effect of metal fiber from recycled tires fiber on (**a**) indirect tensile stiffness modulus (**b**) anisotropy.

Figure 10a shows that the indirect tensile stiffness modulus (ITSM) decreases with increasing temperature and with increase in mixture fiber contents. The physical phenomenon that explains these results is the temperature dependency of bitumen visco-elasticity, i.e., a loss of bitumen viscosity and modulus when temperature increases. Figure 10a shows that the addition of metal fibers from recycled tires decreases the ITSM of mixtures. The average ITSM of the four testing temperatures for the mixtures without fibers was 4865 MPa. Conversely, the average ITSM considering the four testing temperatures and all fiber contents was 2786 MPa. Figure 10b shows all the ITSM measured on the test specimens in the longitudinal (A-A’) and orthogonal (B-B’) directions, respectively. Figure 10b shows that ITSM in A-A’ and B-B’ directions were similar almost for all the specimens tested. In other words, the results indicate that including metal fibers recycled from tires does not induce anisotropy in the axis perpendicular to A-A’ and B-B’. This behavior has been reported in previous research in mixtures with crack-healing capabilities.

**Figure 11:**
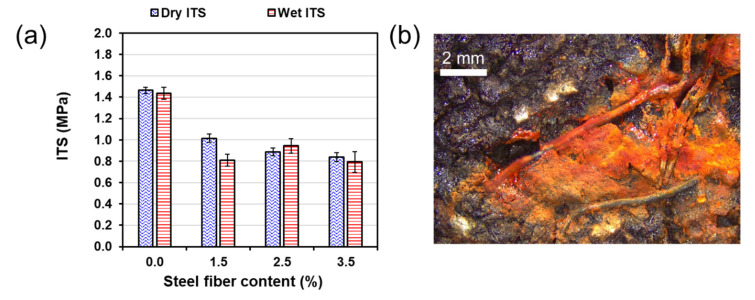
(**a**) Effect of steel fiber on indirect tensile strength, and (**b**) corrosion of metal fibers after soaking. The fibers were not coated with bitumen.

The indirect tensile strength (ITS) results (Figure 11a) show consistency with ITSM. The highest dry ITS of 1.46 MPa was obtained in asphalt samples without steel fiber. The average dry ITS for the mixtures with fiber content of 1.5%, 2.5%, and 3.5% was 1.01 MPa, 0.89 MPa, and 0.84 MPa, respectively, indicating that ITS decreases with increasing fiber content. As expected, wet ITS was lower than dry ITS for asphalt mixtures with 0.0%, 1.5%, and 3.5% fiber content. However, for 2.5% fiber content wet ITS were higher than dry ITS. This result is explained by the lower air voids content of asphalt mixtures with 2.5% fiber content (Figure 4); which was lower than the other mixtures with fiber. The lower air voids content reduces the moisture susceptibility in asphalt mixtures making more difficult water penetration during the 72 h soaking. A common measure of ITS water sensitivity is the indirect tensile strength ratio, which is the ratio between wet ITS and dry ITS. Overall, all the measured ITSR was within acceptable values (>80%) for asphalt mixtures, which has also been observed in previous studies [51].

After soaking, rust was observed on metal fibers that were exposed to water (Figure 11b). Prior to water soaking, there were no signs of rust on the asphalt samples surface. The dimensions of the fibers and air voids content explain these results. Recently, Li et al [52] studied the effect of moisture on metallic fibers used in asphalt concrete with induction properties. The results showed that there was no corrosion inside samples mainly because the bitumen of the mixture coated and protected the steel fibers from water. The average dimensions of the fibers in Li et al.’s (2020) research were 4.2 mm in length and 0.07–0.13 mm. In the current research, the length of the fibers ranged from 11 to 40 mm, and the average diameter was 0.256 mm, making more difficult the coating with bitumen (Figure 11b).

## 4. Conclusions

Waste tires pose a substantial environmental hazard worldwide. Therefore, finding avenues for their reuse and recycling are sought. Although there has been substantial effort to reuse crumb rubber in asphalt pavements, little effort has been made to recycle the substantial waste metal fibers from tires. This work presented the physical, thermophysical, and mechanical properties of asphalt mixtures containing different contents of metallic fiber obtained from recycled tires. Based on the results, the following conclusions have been obtained:

- In general, an increase in the fiber contents increases the air voids content and slightly reduces the bulk density of the mixtures.
- Mixing and compaction was more difficult for higher fiber contents, which is attributed to less space for the bitumen to freely flow and fill the voids of the mixtures.
- Using the computed tomography technique, it was possible to identify clustering of the fibers and that more fibers resulted in more clustering. Furthermore, it was possible to identify the orientation of the fibers that tend to orient towards a horizontal plane, i.e. perpendicular to the compacting force and with the intensity increasing with fibers content. This anisotropy would certainly have an effect in the mechanical performance of the material.
- Samples with metallic fibers presented an electrically conductive behavior; the average electrical resistivity decreased with the increase of the fiber content, thus increasing the electrical conductivity of samples.
- The addition of metallic fibers did not affect the average thermal conductivity with respect to the reference mixture significantly.
- Fiber content had a direct effect on the indirect tensile stiffness modulus (ITSM) that decreased with increasing temperature for mixtures and with increase in fiber contents.
- ITS decreases with increasing fiber content; however, all the measured ITSR values were within acceptable limits (>80%). Some fibers were not coated with bitumen; hence, fiber dimensions should be optimized to increase bitumen coating, reducing the risk of corrosion.
- Overall, the study shows that asphalt mixtures with metallic fiber from waste tires have the potential to be used in asphalt mixtures with crack-healing purposes.

## Figures and Tables

**Table 1:** Particle size distribution of aggregates used.

Size (mm)		Aggregate Fractions		Aggregate Combination
	19 mm	12.5 mm	Crushed Dust	
19	100	100	100	100
12.5	36	100	100	84
10	1	77	100	71
5	1	5	90	47
2.5	0	1	60	31
0.63	0	0	30	14
0.315	0	0	22	10
0.16	0	0	16	7
0.075	0	0	11	5
